# Engineering Degradation Rate of Polyphosphazene-Based Layer-by-Layer Polymer Coatings

**DOI:** 10.3390/jfb15020026

**Published:** 2024-01-23

**Authors:** Jordan Brito, Junho Moon, Raman Hlushko, Aliaksei Aliakseyeu, Alexander K. Andrianov, Svetlana A. Sukhishvili

**Affiliations:** 1 Department of Materials Science & Engineering, Texas A&M University, College Station, TX 77840, USA; jordanbrito@tamu.edu (J.B.);; 2 Institute for Bioscience and Biotechnology Research, University of Maryland, Rockville, MD 20850, USA; 3 Artie McFerrin Department of Chemical Engineering, Texas A&M University, College Station, TX 77843, USA

**Keywords:** degradation, hydrolysis, polymer coatings, layer-by-layer, polyelectrolyte multilayers, polyphosphazenes

## Abstract

Degradable layer-by-layer (LbL) polymeric coatings have distinct advantages over traditional biomedical coatings due to their precision of assembly, versatile inclusion of bioactive molecules, and conformality to the complex architectures of implantable devices. However, controlling the degradation rate while achieving biocompatibility has remained a challenge. This work employs polyphosphazenes as promising candidates for film assembly due to their inherent biocompatibility, tunability of chemical composition, and the buffering capability of degradation products. The degradation of pyrrolidone-functionalized polyphosphazenes was monitored in solution, complexes and LbL coatings (with tannic acid), providing the first to our knowledge comparison of solution-state degradation to solid-state LbL degradation. In all cases, the rate of degradation accelerated in acidic conditions. Importantly, the tunability of the degradation rate of polyphosphazene-based LbL films was achieved by varying film assembly conditions. Specifically, by slightly increasing the ionization of tannic acid (near neutral pH), we introduce electrostatic “defects” to the hydrogen-bonded pairs that accelerate film degradation. Finally, we show that replacing the pyrrolidone side group with a carboxylic acid moiety greatly reduces the degradation rate of the LbL coatings. In practical applications, these coatings have the versatility to serve as biocompatible platforms for various biomedical applications and controlled release systems.

## 1. Introduction

The field of drug delivery has witnessed remarkable progress over recent decades, catalyzed by the development of innovative materials and technologies [1,2,3,4,5]. Among these advances, degradable layer-by-layer (LbL) polymer coatings have emerged as a transformative approach, offering the potential of precise control over drug release kinetics and versatile options for incorporation of bioactives [6,7,8,9,10,11,12]. These coatings, characterized by their conformal, stratified architecture, have garnered significant attention for their potential to revolutionize drug delivery systems. The properties of the coatings can be designed through rational selection of the aqueous assembly conditions, including pH, temperature, and salt concentration [13,14,15,16]. Of particular interest is the ability to fine-tune the degradation rates of these LbL polymer coatings, a feature that has far-reaching implications for the controlled release of therapeutics [17,18].

Degradable LbL coatings offer a versatile platform to customize the release profiles of encapsulated drugs. Previous work in degradable LbL coatings has often focused on the family of poly(β-amino esters), which were assembled with therapeutic molecules such as plasmid DNA, siRNA, enzymes, antibiotics, and growth factors [19,20,21,22,23,24,25,26,27,28]. These LbL systems using poly(β-amino esters) are cationic and degrade from hours to months, with some control of the degradation rate via synthetic modification of the backbone [29,30]. In this work, we selected to incorporate polyphosphazenes into our LbL systems due to their significant advantages in chemical versatility. The backbone, consisting of phosphorous and nitrogen molecules, can degrade hydrolytically into buffering, biocompatible phosphate and ammonium salt products [31,32]. The two side groups attached to each phosphorus atom can be modified extensively, allowing for a wide range of properties and functionalities [33]. Conjugation of poorly soluble drugs to the polyphosphazene backbone is also possible, further highlighting the necessity of tunable degradation [34,35,36,37,38]. Polyphosphazenes also show remarkable biocompatibility, both alone and within LbL coatings, making them an ideal candidate for biomedical applications [39,40].

Notably, the ability to select the hydrophobicity/hydrophilicity of the side groups allows the engineering of a fine-tuned degradation rate of the polyphosphazene [32,41,42,43]. However, although the degradation of many versatile polyphosphazenes has been extensively studied, there are no reports of investigations of their degradation in LbL films. We hypothesized that ‘locking’ polyphosphazene molecules in specific conformations in the films through LbL assembling at a particular solution condition (such as pH) can open an opportunity for controlling the film degradation rate via assembly conditions. Furthermore, there are no studies comparing the degradation of polyphosphazenes in solution-state versus solid-state. To bridge this research gap, we sought to investigate the degradation of a pyrrolidone-substituted polyphosphazene, poly[di(2-(2-oxo-1-pyrrolidinyl)ethoxy)phosphazene] (PYRP), dissolved in solution, in complexes, and assembled at a surface in a LbL coating. Additionally, the release of the pyrrolidone side group, N-(2-hydroxyethyl)-2-pyrrolidone, from PYRP upon degradation may be beneficial for transdermal drug delivery systems, as it was reported to be a skin permeation enhancer [44]. Skin permeation enhancers play a pivotal role in improving drug diffusion across the stratum corneum by modifying its barrier properties without causing significant damage [45,46,47]. We envision that polymers releasing N-(2-hydroxyethyl)-2-pyrrolidone can be useful for applications in coatings on bandages to accelerate wound healing, on dermal patches to deliver bioactives [48,49], or for the controlled release of drugs, with the added advantage of releasing skin-permeating enhancers as a byproduct of degradation.

To our knowledge, this study is the first to compare the degradation of a polymer dissolved in solution to the degradation of hydrogen-bonded complexes and hydrated polyelectrolyte multilayers. We also compare the degradation of the PYRP LbL films with a polyphosphazene with hydroxybenzoic acid side groups, poly[di(carboxylatophenoxy)phosphazene] (PCPP) to investigate the effect of side groups on the degradation of LbL. This investigation reveals the differences in the degradation mechanism of solution-state and solid-state systems which is valuable for future criterion of bioactive-releasing technology [50].

In this work, we assemble PYRP with a model hydrogen-bonding molecule, tannic acid (TA) to form LbL coatings, and quantify the degradation in solution over time. Along with being a model molecule, TA also has clinical relevance as a high molecular weight polyphenol, with many hydroxyl groups available for hydrogen-bonding, and many biological benefits including high antioxidant activity and anti-cancer, antibacterial, antiviral, and antifungal properties [51]. The *pK_a_* of TA is reported to be ~8.5, allowing hydrogen bonds to be stable even near neutral pH [52]. By adjusting the pH of the assembly conditions or degradation conditions, we show that we can control the rate of degradation of the hydrogen bonded LbL films. From infrared (IR) analysis of the LbL films, we also evaluate the degradation rate of the polyphosphazene backbone and the cleavage rate of the side group, providing a hypothesis for the mechanism of degradation of PYRP in the LbL system. This mechanism is also compared to solution-state degradation of PYRP and PYRP/TA complexes. Taken together, this work provides a basis for an understanding of solution-state degradation versus solid-state LbL degradation, while showing for the first time that the degradation of polyphosphazene-based LbL coatings can be controlled through rational design of bonding between the assembly partners and assembly conditions.

## 2. Materials and Methods

### 2.1. Materials

Sodium phosphate monobasic dihydrate, hydrochloric acid (Sigma-Aldrich, Saint Louis, MO, USA), sodium hydroxide, and sulfuric acid (VWR International, Radnor, PA, USA) were used as received. Branched poly(ethylenimine) (BPEI, *M_w_* = 750 kDa, Sigma-Aldrich, Saint Louis, MO, USA), tannic acid (TA, Sigma-Aldrich, Saint Louis, MO, USA), poly(diallyldimethylammonium) chloride (PDADMAC, *M_w_* = 75 kDa, PSS Polymer Standards Service GmbH, Mainz, Germany), and poly(vinylpyrrolidone) (PVP, *M_w_* = 360 kDa, Alfa Aesar, Ward Hill, MA, USA) were used as received. Poly[di(carboxylatophenoxy)phosphazene] (PCPP, *M_w_* = 200 kDa) and poly[di(2-(2-oxo-1-pyrrolidinyl)ethoxy)phosphazene] (PYRP, *M_w_* = 340 kDa) were synthesized as described previously [53]. Silicon wafers (University Wafer, Inc., South Boston, MA, USA; ⟨100⟩ orientation, undoped, 0–100 Ω·cm resistivity) and AT-cut quartz crystals were used as substrates for multilayer deposition. Ultrapure Milli-Q water (MilliporeSigma, Burlington, MA, USA) with a resistivity of 18.2 MΩ·cm was used in all LbL experiments.

### 2.2. Dynamic Light Scattering (DLS) 

DLS measurements were performed using a Malvern Zetasizer Nano ZS (Malvern Instruments Ltd., Worcestershire, UK) equipped with a 532 nm laser and a back-scattering detector at an angle of 173°. Data were recorded and processed in Zetasizer Software 7.10 (Malvern Instruments Ltd., Worcestershire, UK). Samples were filtered through poly(vinylidene fluoride) (PVDF) Millex syringe filters (EMD Millipore, Billerica, MA, USA) with a pore size of 0.22 µm prior to measurements. DLS measurements were performed at 25 °C using the BRAND^®^ (BRAND GMBH + CO KG, Wertheim, Germany) disposable ultra-micro cuvettes with plastic caps for polymer and complex formulations containing 0.5 mg/mL PYRP in 50 mM phosphate buffer. Each measurement was averaged from 12 scans.

### 2.3. Residual Molecular Weight 

Prior to measurements, the size-exclusion column and cellulose membrane were calibrated using GPC/SEC PAA standards (American Polymer Standards Corp., Mentor, OH, USA). Residual molecular weight was calculated according to the equation:

*Residual M_w_* (%) = *M_i_*/*M*_0_ × 100%

where *M*_0_ is the initial molecular weight and *M_i_* is the molecular weight of PYRP on day *i* of the degradation experiment.

### 2.4. Size-Exclusion Chromatography (SEC) 

SEC analysis of the polymer was conducted using an Agilent 1260 Infinity II Binary LC system (Agilent, Santa Clara, CA, USA) equipped with a G7112B binary pump, G7167A Multisampler, G7116A multicolumn thermostat, G7117C diode array detector, G7121A fluorescence detector, and TSKgel GMPW size-exclusion column (Tosoh Bioscience, LLC., Tokyo, Japan). Phosphate buffered saline (PBS) with 10 vol% acetonitrile was used as a mobile phase.

### 2.5. Asymmetric Flow Field Flow Fractionation (AF4) 

AF4 was performed using a Postnova AF2000 MT series (Postnova Analytics GmbH, Landsberg, Germany). The system was equipped with two PN1130 isocratic pumps, PN7520 solvent degasser, PN5120 injection bracket, and a UV–Vis detector (SPD-20A/20AV, Shimadzu Scientific Instruments, Columbia, MD, USA). A regenerated cellulose membrane with a molecular weight cutoff of 10 kDa (Postnova Analytics GmbH, Landsberg, Germany) and a 350-μm spacer were used in a separation micro-channel employing both laminar and cross flows of an eluent—PBS (pH 7.4). The collected data were processed using the AF2000 software (Postnova Analytics GmbH, Landsberg, Germany).

### 2.6. Layer-by-Layer (LbL) Film Deposition 

To prepare for LbL deposition, large-size silicon wafers lightly scratched into 1 cm × 1 cm sections (for ellipsometric and FTIR measurements) and quartz crystals (for QCM measurements) were cleaned by overnight exposure to UV light and placed in a sulfuric acid bath for 40 min to clean and introduce silanol groups to the surface. Then, the substrates were thoroughly rinsed with Milli-Q water to remove all remaining sulfuric acid, and dried with nitrogen gas before deposition. All solutions used for LbL deposition, i.e., phosphate buffer (PB) rinses (0.01 M), PYRP, TA, PCPP, and PDADMAC solutions (all 0.2 mg/mL in 0.01 M PB) were adjusted to pH 3, 5, or 6.8 by additions of 0.1 or 0.01 M NaOH or HCl. All deposition solutions were stored in the refrigerator overnight before deposition to allow them to dissolve while preventing premature degradation. All solutions were brought to room temperature prior to deposition.

Prior to the deposition of LbL films, the substrates were primed with a monolayer of BPEI (0.2 mg/mL in Milli-Q water, adjusted to pH 9) for 15 min and rinsed with 0.01 M PB at the specified pH of the deposition (i.e., pH 3, 5, or 6.8). The BPEI-primed substrates were then placed back-to-back (to avoid deposition of polymer on the back of the substrate) and held together with tweezers for LbL deposition using a dipping robot (DR-3 Table Top Dipping Device, Riegler & Kirstein GmbH, Potsdam, Germany). For TA/PYRP films, the wafers were first dipped into TA solution for 5 min and withdrawn at a speed of 1 cm/s, followed by two PB rinsing steps (10 fast dips in both rinse beakers), then dipped into PYRP solution for 5 min and withdrawn at a speed of 1 cm/s, followed by two PB rinsing steps (10 fast dips in both rinse beakers). This process was repeated until the LbL films reached 400 nm, as determined by ellipsometric and QCM measurements. A similar procedure was used to deposit PCPP/PDADMAC films, with PCPP solution in place of the TA solution, and PDADMAC in place of the PYRP solution. The multilayers were capped with PCPP in PCPP/PDADMAC systems and with PYRP in the PYRP/TA systems.

### 2.7. Spectroscopic Ellipsometry 

After each bilayer, the thickness and refractive index of the LbL films were characterized using a M-2000 automated-angle spectroscopic ellipsometer (J.A. Woollam Co., Inc., Lincoln, NE, USA). Dry film measurements were collected at three different locations and at four incidence angles: 45, 55, 65, and 75°. To fit the ellipsometric data, the dry polymer film on the Si wafer was treated as a graded layer of Cauchy material with the thickness *d* atop a silicon and native oxide layer. Fitting coefficients and thickness *d* were fitted simultaneously. Liquid cell measurements, used to measure the swelling of the films, were collected at 75° until a constant swollen thickness was reached (~30 min). Prior to filling the cell with pH adjusted PB, the dry film thickness was measured in the cell for an accurate calculation of the swollen thickness. 

### 2.8. Quartz Crystal Microbalance (QCM) Measurements 

The QCM device (QCM200, Stanford Research Systems, Inc., Sunnyvale, CA, USA) consists of an AT-cut quartz crystal with a resonant frequency of 5 MHz, a diameter of 25.4 mm, and Cr/Au electrodes. It should be noted that during LbL deposition, as described above, two QCM crystals were placed back-to-back to ensure that the film was exclusively deposited on the front side of each electrode. The prepared QCM devices were used for the degradation study. The frequency changes during deposition/degradation of the films were monitored, which can be correlated with the absorbed/desorbed mass on the QCM crystal surface according to the Sauerbrey equation:

∆*m* = −*C* × ∆*f*/*n*

where ∆*m* is the mass change, ∆*f* is the frequency shift, *n* is the overtone number, and *C* is a crystal-specific constant which is equivalent to 17.7 ng/cm^2^∙Hz for a 5 MHz AT-cut quartz crystal.

### 2.9. Fourier Transform Infrared (FTIR) Spectroscopy 

A Bruker Tensor II spectrometer equipped with a mercury cadmium telluride detector (Bruker Optik GmbH, Ettlingen, Germany) was used for collecting transmission FTIR spectra. The spectra were accumulated by 96 scans in a spectral range of 500–4000 cm^−1^ at a resolution of 2 cm^−1^.

To analyze the spectral changes during degradation of the LbL films, the films deposited on undoped silicon wafers were dried with nitrogen gas upon removal from the degradation PB, and the infrared spectrum was collected in transmission mode every 3–4 days. For peak identification, a monolayer of PCPP, PDADMAC, PYRP, or TA (dissolved in ultrapure water) was deposited on a diamond ATR crystal by solution evaporation, and an infrared spectrum was collected for each under a constant stream of nitrogen gas to inhibit water absorption from the air. Relevant peaks were identified and deconvoluted in ImageJ. The peak heights and wavenumbers were quantified for each time interval.

### 2.10. Optical and Scanning Electron Microscopy 

Optical microscope images of the as-deposited and degraded LbL films on silicon substrates were collected using an Olympus DXS500 microscope (Olympus Deutschland GmbH, Hamburg, Germany) with 10× and 50× magnification lenses. Scanning electron microscopy (SEM) images were collected using a Helios G4 DualBeam microscope (Thermo Fisher Scientific Inc., Waltham, MA, USA) at 1 kV to observe the morphology of the as-deposited and degraded LbL films on silicon substrates without needing any metal sputter-coating.

## 3. Results

### 3.1. Layer-by-Layer Assembly of PYRP/TA at pH 3, 5, and 6.8

**Figure 1:**
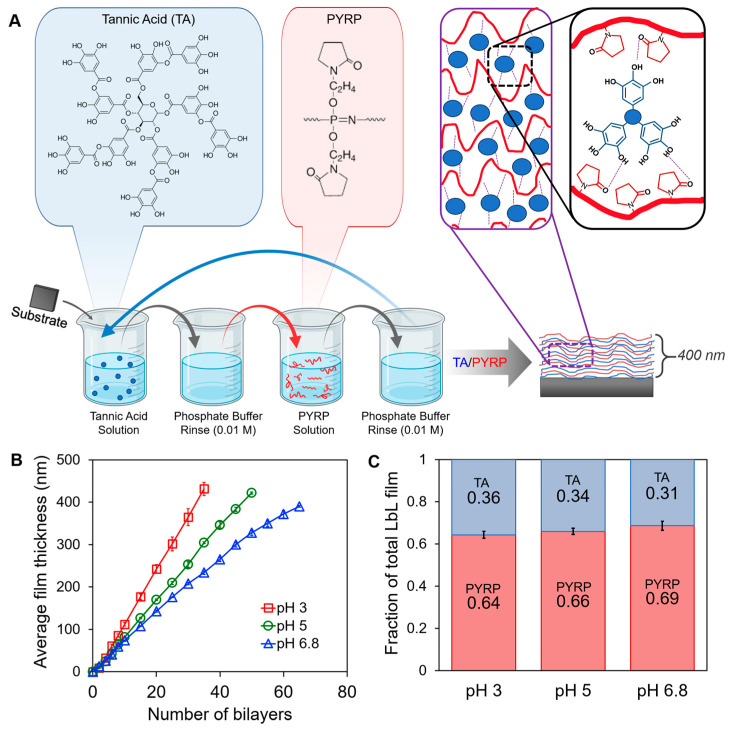
(**A**) Graphic representation of PYRP/TA LbL assembly and hydrogen bonding between the pyrrolidone groups in PYRP and gallol groups in TA. The LbL schematic shows the sequential dipping of a substrate into TA solution, rinse solutions, PYRP solution, and rinse solutions to form one bilayer. This sequence is repeated until our desired thickness, 400 nm, is reached. (**B**) LbL growth curve of PYRP/TA at pH 3, 5, and 6.8, as determined by spectroscopic ellipsometry. (**C**) Fraction of TA and PYRP within the PYRP/TA LbL films assembled at pH 3, 5, and 6.8.

PYRP and TA multilayers (denoted PYRP/TA) were constructed via hydrogen bonding between subsequent monolayers of pyrrolidone-containing PYRP and gallol-containing TA (Figure 1A) up to 400 nm for enhanced sensitivity in IR characterization. Due to the nature of hydrogen-bonding, the PYRP/TA assembly is highly affected by the assembly pH. To assemble stable hydrogen-bonded films, we chose acidic assembly conditions, pH 3, pH 5, and pH 6.8, where TA has low to no negative charge. The selection of these pH values also facilitated accelerated degradation when degrading the samples in matched pH conditions. Considering a *pK_a_* of ~8.5 [52], TA has approximately 2% ionization at pH 6.8 and virtually no ionization at pH 3 and 5. In the condensed state of the LbL film, the 2% ionization can have significant effects on the growth curve and degradation. The effect of ionization is clear in the film growth curves (Figure 1B), where assembly at pH 6.8 shows slower growth compared to pH 3 and 5. The slight ionization inhibits the formation of hydrogen bonds, slowing the growth of the layers. The slower growth of films assembled at pH 6.8 than in more acidic conditions was also confirmed via quartz crystal microbalance (QCM) measurements (Supplementary Materials, Figure S1). Within the films, the mass ratio between TA and PYRP is about 1:2 (Figure 1C), as determined by spectroscopic ellipsometry, with negligible differences between assembly pH.

### 3.2. Degradation of PYRP

To interpret distinct features of PYRP/TA degradation, both in solution and as part of LbL coatings, the kinetics and molecular mechanism of hydrolytic breakdown of PYRP alone was studied first. As reported previously, the mechanistic pathway of PYRP hydrolysis in aqueous solution includes side group cleavage and the formation of unstable phosphazane functionalities, which results in a subsequent breakdown of the main chain [53]. Accordingly, the kinetics of polymer degradation in solutions of various pH at 50 °C were studied via SEC, DLS, and AF4 by monitoring both the detachment of side groups and changes in the molecular weight of the polymer. We selected acidic pH values and 50 °C for all further experiments to accelerate the rate of degradation of the systems, particularly the slowly degrading LbL systems.

**Figure 2:**
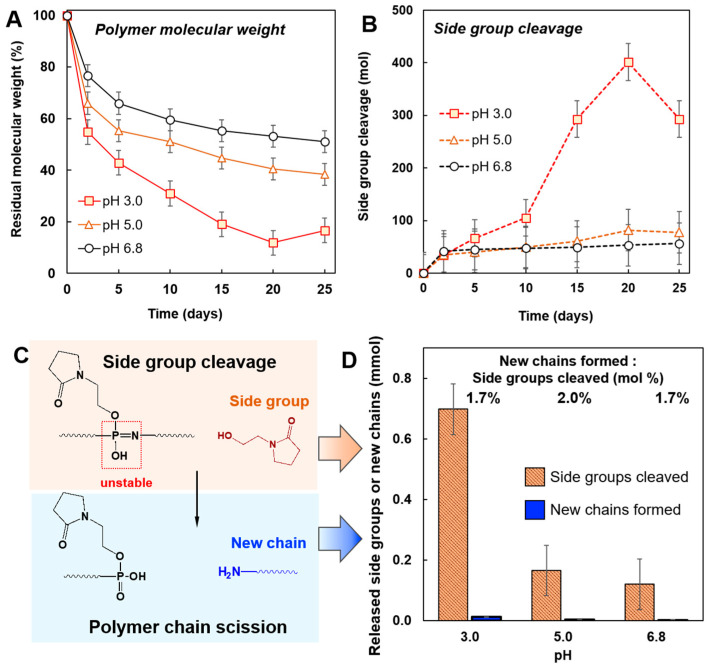
Degradation of PYRP at pH 3.0, pH 5.0, or pH 6.8 and 50 °C. (**A**) Residual molecular weight and (**B**) the cleavage of side groups per polymer chain as the result of polymer degradation. (**C**) Schematic representation of the hydrolysis of PYRP showing the cleavage of a side group and the formation of an unstable phosphazane group, followed by scission of the polymer chain. (**D**) Comparison of the side group cleavage and formation of new macromolecular chains as a result of chain scission during a 25 day period (0.5 mg/mL PYRP; 0.01 M phosphate buffer, SEC data). The units of both side groups cleaved and new chains formed are in mmol, and the ratio between them is in mol%.

The loss in the molecular weight of the polymer was most pronounced at pH 3.0—approximately 80% over the 25-day period—with the rate of degradation slowing down as the pH of solution increases, i.e., 60% loss at pH 5.0 and 50% loss at pH 6.8 (Figure 2A and Supplementary Materials, Figure S2). Independent measurements of hydrodynamic diameter of PYRP by DLS (Supplementary Materials, Figure S3) and its molecular weight by AF4 (Supplementary Materials, Figure S4) were in line with the SEC findings. Such pH dependence agrees with the previously reported acid-catalyzed mechanism of polyphosphazene degradation in aqueous solutions [53,54]. The total amount of the side group (N-(2-hydroxyethyl)-2-pyrrolidone) cleaved over the 25-day degradation experiment was near 300 molecules per polymer chain at pH 3.0 vs. approximately 80 or 60 molecules at pH 5.0 and 6.8, respectively (Figure 2B). Figure 2C shows the schematics of the hydrolytic pathway, in which cleavage of the side group and the formation of the phosphazane functionality is followed by the polymer backbone scission. Comparison of the amount of side groups cleaved with the number of new chains formed as the result chain scission at 25 days reveals that only 1.7–2% of the side group cleavages result in the breakdown of the polymer chain (Figure 2D).

### 3.3. Degradation of PYRP/TA Complexes in Solution and LbL Coatings

**Figure 3:**
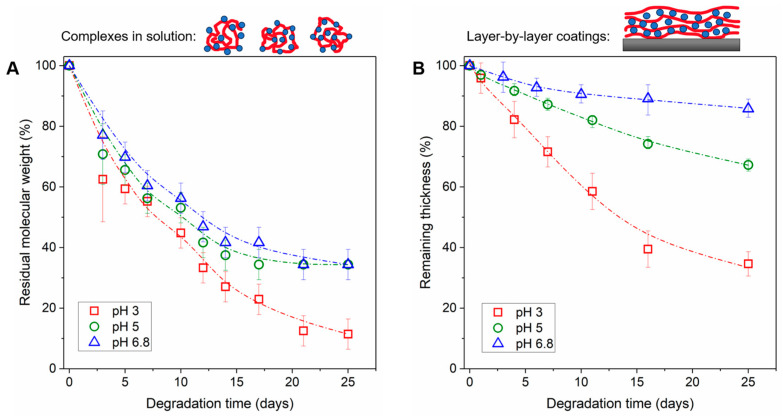
Degradation of (**A**) PYRP/TA complexes (1:20 TA:PYRP mass ratio) and (**B**) 400 nm PYRP/TA LbL films (1:2 TA:PYRP mass ratio, as seen in Figure 1C) upon exposure to 0.01 M phosphate buffer at a matched pH of assembly (i.e., pH 3, pH 5, or pH 6.8) and 50 °C. Complexes were monitored via AF4, and LbL films via spectroscopic ellipsometry.

The solution-state degradation of PYRP/TA complexes was explored by AF4, as the analysis by SEC was adversely impacted by the non-specific interactions of TA with the column, and DLS data were convoluted by the formation of supramolecular structures. Note that due to precipitation of PYRP/TA complexes formed with equal amounts of TA and PYRP, the ratio of TA to PYRP was kept at a 1:20 (TA:PYRP) mass ratio (4 repeat units of pyrrolidone per one OH group in TA), i.e., it was much lower than in LbL films. Comparison of degradation results for PYRP and PYRP/TA complexes revealed essentially identical profiles (Figure 3A, Supplementary Materials, Figures S5 and S6), indicating the negligible effect of TA on the polymer degradation rate.

In LbL films, we first compared the rate of degradation without the effect of pH mismatch and equilibration—by exposing the films to the same pH as the assembly pH. The films assembled at pH 3 and degraded at pH 3 showed the most rapid degradation, as shown by ellipsometry, with approximately 70% of the film degrading in 25 days (Figure 3B). This result is consistent with the solution-state data showing acid catalysis of PYRP. The films assembled at pH 5 and degraded at pH 5 showed slower degradation, losing ~30% of the original thickness in 25 days. As expected, the slowest degradation occurred with the films near neutral pH, assembled at pH 6.8 and degrading at pH 6.8.

Optical images and scanning electron micrographs showed that all as-deposited films started with a uniform surface but exhibited different morphologies after the 25-day degradation (Supplementary Materials, Figures S7 and S8). The film assembled at pH 3 and degraded at pH 3 displayed a rough surface with some pore formation after 25 days, possibly because of the higher rate of degradation. The film assembled at pH 5 and degraded at pH 5 still showed a uniform surface, and the film assembled at pH 6.8 and degraded at pH 6.8 showed some wrinkling on the surface after 25 days.

### 3.4. Molecular Mechanism of LbL Coating Degradation

**Figure 4:**
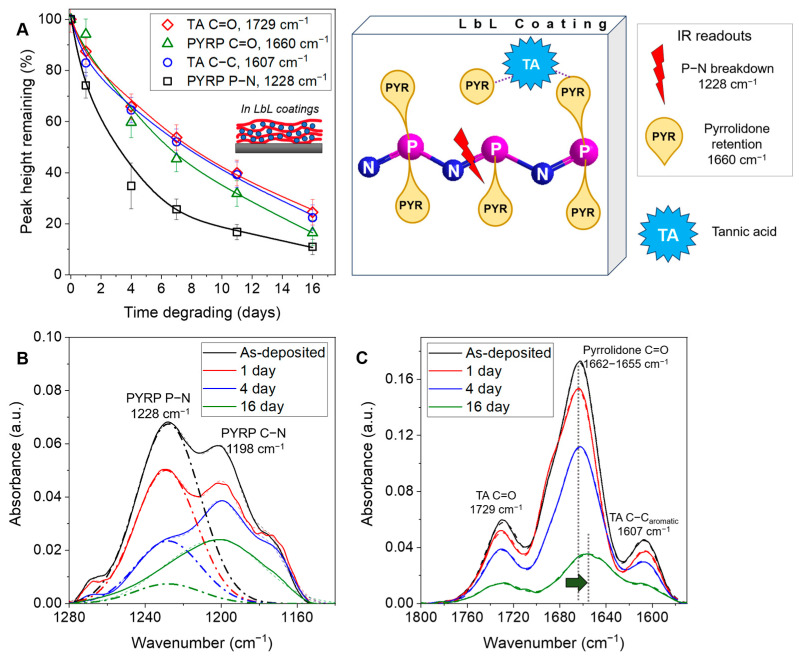
(**A**) Peak height reduction of the pyrrolidone, TA, and phosphazene groups in the TA/PYRP films assembled at pH 3 and degraded in 0.01 M phosphate buffer at pH 3, 50 °C. Schematic shows the preferential hydrolysis of P-N backbone bonds over release of the N-(2-hydroxyethyl)-2-pyrrolidone side group. Experimental FTIR data (solid line), fitted data (dashed line), and relevant deconvoluted peaks (bold dot-dash line) are shown for the 1140–1280 cm^−1^ (**B**) and 1570–1800 cm^−1^ (**C**) regions.

By further investigation of the TA/PYRP multilayers assembled and degrading at pH 3, we aimed to establish whether PYRP degradation in LbL coatings follows the same mechanism of chain scission due to the cleavage of the side groups. As discussed previously, in solution, we observed a much higher rate of side group cleavage than chain scission. However, we hypothesized that while in solution experiments side group cleavage is accompanied by immediate release of N-(2-hydroxyethyl)-2-pyrrolidone, side group cleavage and actual release of N-(2-hydroxyethyl)-2-pyrrolidone in solution can be decoupled in the film as the release of the side group product from the film can be delayed by hydrogen-bonding with TA. Using transmission FTIR, we tested this hypothesis by monitoring the retention of relevant chemical functional groups within the film over time, i.e., pyrrolidone in the side group, phosphazene in the backbone, and TA groups. As shown in Figure 4A, the rapid reduction of the 1228 cm^−1^ P-N stretching vibrational peak associated with the PYRP backbone signified a fast scission of the polyphosphazene backbone and a higher retention of the pyrrolidone side group over time (quantified from the intensity of amide I peak at ~1660 cm^−1^). Notably, the reduction in the P-N backbone peak (Figure 4B) was also faster than the change in film thickness (Figure 3B, red curve), suggesting that the cleaved fragments of the degrading PYRP chains were retained within the LbL coating due to binding with TA. A shift of the amide I peak of the pyrrolidone moiety of PYRP from 1662 cm^−1^ to 1655 cm^−1^ (Figure 4C and Supplementary Materials Figure S9) suggests that upon film degradation the segments of strongly hydrogen-bonded PYRP are held within the film as weaker hydrogen-bonded segments are cleaved and released. Furthermore, the retention of TA in the film over time (represented by the peaks at 1729 cm^−1^ & 1607 cm^−1^) was nearly equal to the retention of the pyrrolidone side groups, signifying the combined release of both the model bioactive molecule TA and the skin permeation-enhancing N-(2-hydroxyethyl)-2-pyrrolidone. The peak at 1198 cm^−1^, assigned to the stretching vibrations of C-N bond in the pyrrolidone group, also followed the same trend (Supplementary Materials, Figure S10).

The PYRP/TA LbL films assembled/degraded at pH 5 and pH 6.8 showed slower rates of degradation (Figure 3B), so trends in the reduction of their IR peaks were not as pronounced. In the pH 5/pH 5 films, the rate of peak reduction was consistent with the change in thickness, losing ~30% of the original measurements after 25 days (Supplementary Materials, Figure S11). In the pH 6.8/pH 6.8 films, the likely oxidation of TA into a quinone derivative [55] may have caused the deformation of the IR spectra and initial peak losses, but the peak reduction rate nearly matched the change in thickness after 3 days (Supplementary Materials, Figure S12). As a control experiment, we compared the PYRP/TA results to a non-degradable poly(vinyl pyrrolidone) (PVP)/TA LbL system assembled at pH 3 and ‘degrading’ at pH 3, which showed negligible changes in the thickness and IR spectra (Supplementary Materials, Figure S13). This control experiment confirmed that degradation of the PYRP/TA systems occurred due to chemical degradation of PYRP chains, rather than simple erosion due to destabilization of PYRP/TA interactions.

### 3.5. Effect of Initial Assembly pH on Degradation: Assembly pH 3 and 6.8 versus Degradation at pH 5

**Figure 5:**
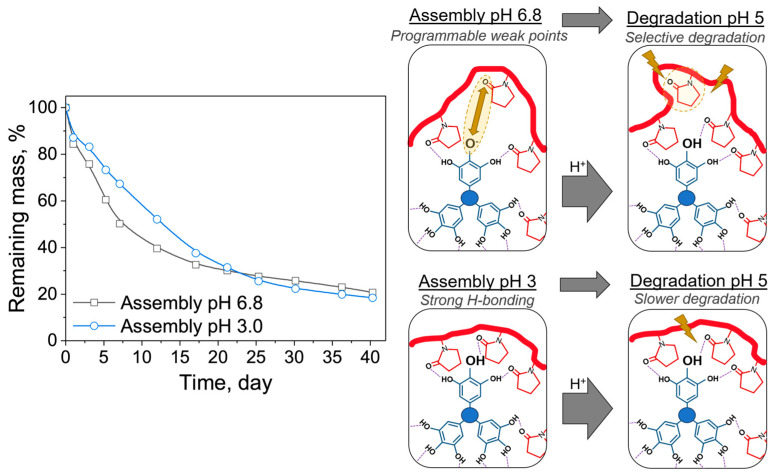
Degradation of PYRP/TA assembled at pH 3 and 6.8 and degraded in 0.01 M phosphate buffer at pH 5 and 50 °C, as determined by QCM. Schematic shows the ‘defects’ in assembly caused by the small amount of ionization at assembly pH 6.8, which can lead to more rapid degradation of the weakly hydrogen-bonded areas around the ‘defect’.

To investigate the effect of assembly pH on film degradation at a fixed pH, we assembled LbL films at pH 3 and pH 6.8 and put them into pH 5 solution for degradation. Measurements were collected after the first 30 min to allow the film to adapt to the initial pH mismatch. As shown in Figure 5, the films assembled at pH 6.8 initially degraded faster than the films assembled at pH 3. This effect also was confirmed via spectroscopic ellipsometry (Supplementary Materials, Figure S14). We hypothesize that this difference in the initial degradation rate is due to programmable ‘defects’ in the interlayer interactions stemming from the initial assembly conditions. As mentioned earlier, TA has ~2% charge near neutral pH, yet those few charges can cause significant electrostatic repulsion within the densely packed multilayers and can potentially create a more hydrated microenvironment for film degradation. Thus, these ‘defects’ are likely to be more hydrolytically labile than the groups stabilized by hydrogen bonds, leading to rapid initial degradation, as shown in the schematic in Figure 5. Slight differences between the rate observed with QCM versus ellipsometry probably resulted from substrate effects, as the film was deposited on a gold-coated quartz crystal for QCM measurements and on a silicon wafer for ellipsometric measurements.

### 3.6. Effect of Polyphosphazene Side Group on the Degradation of LbL Coatings: PYRP/TA versus PCPP/PDADMAC

**Figure 6:**
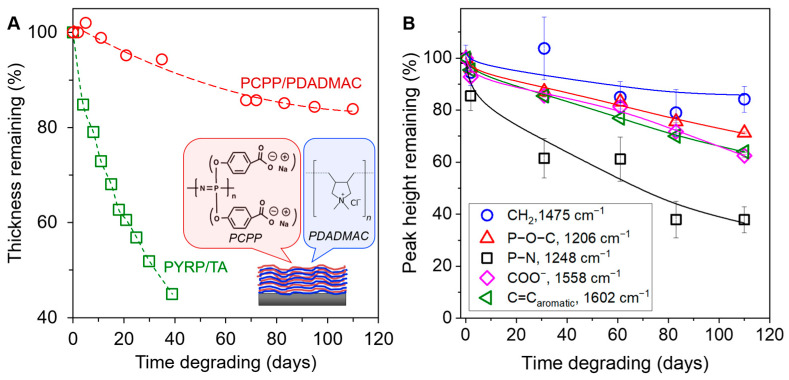
(**A**) Degradation of PYRP/TA compared to PCPP/PDADMAC films (both assembled at 6.8 and degraded in 0.01 M phosphate buffer at pH 5, 50 °C) as determined by spectroscopic ellipsometry. The schematic shows the chemical structures of the alternating PCPP and PDADMAC layers. (**B**) IR peak height reduction of the PDADMAC (CH_2_), alkoxybenzoic acid (P–O–C, COO^−^, and C=C_aromatic_) and phosphazene (P–N) groups in the PCPP/PDADMAC films.

The structural diversity of polyphosphazenes is mainly attributed to the macromolecular substitution synthetic route. To investigate the effect of side group on the rate of degradation, we assembled an anionic polyphosphazene with alkoxybenzoic acid side groups, poly[di(carboxylatophenoxy)phosphazene (PCPP), with strong polycation poly(diallyl dimethylammonium chloride) (PDADMAC) and monitored the degradation over 110 days. PCPP is a water-soluble polymer with reported applications in vaccine delivery and microencapsulation [56,57,58]. PCPP shows higher resistance to degradation in solution than other polyphosphazenes with more hydrophilic side groups, such as PYRP [53]. When assembled at pH 6.8 and degraded at pH 5, the PCPP/PDADMAC system degraded at a rate of ~0.15% film thickness per day, in comparison to PYRP/TA degradation at ~1.5% per day (Figure 6A). This tenfold decrease in degradation rate is because of the side group chemistry/hydrophobicity and demonstrates the wide range of degradation rates that can be reached by various combination of polyphosphazenes and binding partners. Additionally, the PCPP/PDADMAC system also showed a preferential cleavage of the phosphazene bond and high retention of the hydroxybenzoic acid side group (Figure 6B), similar to the PYRP/TA LbL system. However, unlike the rapidly degrading PYRP/TA LbL systems, the effect of assembly pH on the degradation rate was negligible in the 110-day period (Supplementary Materials, Figure S15).

## 4. Discussion

In this project, we mostly sought to achieve controlled hydrolytic degradation of polyphosphazenes in LbL coatings and compare the rates of polyphosphazene degradation in solutions and within the film. Our solution-state studies of degradation of PYRP included within PYRP/TA complexes were conducted in lower-acidity solution conditions than previously reported and thus showed higher degradation, but the trend agreed with the reported acid-catalyzed hydrolysis of PYRP. PYRP/TA complexes of a 1:20 TA:PYRP mass ratio (4 repeat units of pyrrolidone per OH group in TA) degraded at a similar rate as the PYRP. When assembled into the LbL coatings, the ratio of TA included in the coatings was much higher than in the PYRP/TA complexes (1:2 TA:PYRP mass ratio, Figure 1C). The PYRP/TA LbL film also showed acid-catalyzed degradation, yet at an even slower degradation rate than the PYRP/TA complexes. When assembled into LbL coatings, the acidic functional groups (i.e., hydroxyl groups in TA) are condensed into nearly 2D layers, which may accelerate the degradation due to end group proximity [50]. However, as seen in our results, the hydrogen bonding between the components also stabilizes the coatings by holding cleaved PYRP chains within the coatings until the interlayer complexes are soluble enough to leave the coating, slowing degradation compared to solution degradation.

**Scheme 1:**
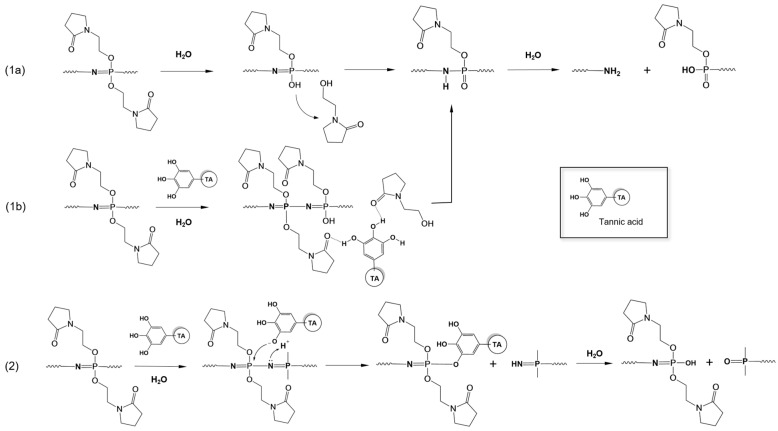
Potential pathways for the solution and solid-state degradation of PYRP in the absence (**1a**) and presence of TA (**1b**,**2**). Scheme (**1a**) occurs in solution, while Scheme (**1b**,**2**) likely occur in the LbL films.

The degradation mechanism of PYRP in solution was previously suggested to follow a well-established pathway [53,59], which includes the cleavage of a side group, protonation of the backbone nitrogen followed by proton migration, formation of the unstable phosphazane group, and eventual chain scission (Scheme 1, pathway 1a). Present results for the pH-dependent solution-state degradation of PYRP, which were explored using a suite of SEC, AF4, and DLS methods, are in line with previous findings. Somewhat unexpectedly, the comparison of SEC-detected moles of side group cleaved to the number of new polymer chains formed through chain scission revealed an approximately fifty-fold lower incidence of the chain scission. This either indicates a relative stability of phosphazane moieties or the ‘unzipping’, i.e., degradation of the polymer from the chain end, which results in the formation of only one macromolecule detectable by SEC, rather than main chain scission [60]. Addition of TA to the PYRP solution (at about 23 TA molecules per PYRP chain) did not change the kinetics of degradation, although the conclusion was made solely on AF4 data due to the limitations of the SEC and DLS methods.

The analysis of PYRP degradation in the TA assembled films revealed some important distinctions with the above observations. Within the film, we saw a slower degradation rate in PYRP/TA LbL films (as determined by change in thickness and mass) than the PYRP and PYRP/TA complexes (as determined by decrease in molecular weight). Additionally, through SEC analysis, we showed that the release of side groups is rapid in solution, but FTIR analysis of LbL films showed a higher retention of pyrrolidone groups. The PYRP/TA LbL film was advantageous for IR analysis due to the stability of the film at the silicon surface—transmission FTIR measurements yielded high resolution spectra throughout the degradation period. The characteristic peaks associated with the backbone and side groups were identified and deconvoluted for quantification of the rate of their disappearance. While a parallel can be drawn between the rate of phosphazene bond breakage in LbL films and the reduction in molecular weight in solution-state (Figure 3), the data on the release of side groups from the soluble PYRP/TA system and PYRP/TA film were distinctly different (Figure 2D and Figure 4A), especially when compared to the rate of chain scission. This can be viewed in the context of the constrained environment, where molecular binding in the film and the resulting local dehydration inhibits the removal of the degradation product. Hydrogen bonds formed between TA molecules and the detached pyrrolidone side groups (Scheme 1, pathway 1b) can further impede the release process. An alternative explanation of this phenomenon can be based on a previously suggested mechanism for acid-catalyzed hydrolysis of polyphosphazenes [61], which involves direct interaction between the polymer and TA molecules and can lead to the degradation of the backbone without the concurrent release of the moiety (Scheme 1, pathway 2). It is also important to note that the change in film thickness occurs slower than the reduction of phosphazene bonds, suggesting that the partially degraded polymer is held within the film by the remaining hydrogen-bonded side groups on the cleaved section. After sufficient cleavages of the backbone, the short-chain polyphosphazene and TA become soluble and release from the LbL film.

An important distinct feature of LbL-assembled PYRP as compared to the water-soluble PYRP counterparts is the ability to alter the degradation rate not only by post-assembly degradation conditions (such as pH and temperature), but also by the film assembly conditions. By altering the intermolecular interactions between the layers in the LbL film via assembly pH, we showed the ability to program ‘defects’ into the multilayers due to the slight ionization of TA, leading to faster initial degradation than a system assembled with neutral TA. This result can be used to engineer the rate of an initial burst release of loaded active molecules, and even to take advantage of the defects to load further bioactives into the film post-assembly.

Finally, the comparison of PCPP/PDADMAC and PYRP/TA showed a significant change in the rate of degradation, from weeks for degradation of the PYRP/TA LbL film to months or possibly years using the PCPP/PDADMAC LbL system. The previous study of degradation of PYRP and PCPP in solution reported dramatic differences in degradation rates of these polymers. Specifically, the molecular weight of PYRP decreased to ~30% of the original after 5 days of degradation of polymer solutions, while the molecular weight of PCPP decreased only to ~80% of the original value (in PBS, pH 7.4, 55 °C) [53]. In solution, PYRP degrades much faster than the more hydrophobic PCPP, and the slow degradation of PCPP in the confined environment of the films can be further reduced, almost reaching the limit of practically non-degradable films. Thus, we show that we can control degradation of polyphosphazenes in a wide range by several ways, including side group chemistry partner and, most importantly, through film assembly conditions. Along with the tunable degradation of LbL films containing cationic poly(β-amino esters) (occurring within hours to months) [19,20,21,22,23,24,25,26,27], LbL films containing hydrogen-bonding and anionic polyphosphazenes (PYRP and PCPP, respectively) expand the toolbox for rationally engineered LbL films with precise degradation rates for drug delivery systems that can load a now-wider range of cationic, anionic, and hydrogen-bonding cargo.

Future work includes quantifying the release of the loaded active molecule, along with the polyphosphazene side group (in this case, N-(2-hydroxyethyl)-2-pyrrolidone) to demonstrate the clinical relevance of the degradable film as a skin-permeation enhancer and drug delivery system. Substitution of a small percent of polyphosphazene side groups with a fluorescent tag may allow accurate quantification of the rate of side group release. Mass spectrometry of PYRP after degradation would also allow confirmation of the degradation products, including release of side groups. Additionally, one limitation of this study is that all degradation experiments were conducted at accelerated conditions (acidic pH and 50 °C); however, many biomedical applications are subjected to physiological conditions (pH 7.4, 37 °C, 0.15 M NaCl) that would reduce the degradation rate. Future work may also include degradation studies under conditions dictated by a specific biomedical application.

## 5. Conclusions

This study of the degradation of PYRP, PYRP/TA complexes, and PYRP/TA LbL coatings achieved multiple novel conclusions that enable a rational engineering of degradation rates for LbL assemblies. The acid-catalyzed hydrolysis of polyphosphazene PYRP was proven for PYRP alone, PYRP/TA complexes, and PYRP/TA LbL films, which, to our knowledge, is the first instance of direct comparison of solution-state degradation to degradation in the solid-state LbL coatings. The degradation of PYRP/TA LbL coatings was explored for LbL films assembled at pH 3 and degrading at pH 3, and the release mechanism of the product of the side group cleavage (N-(2-hydroxyethyl)-2-pyrrolidone) was proposed based on the results of IR analysis. Importantly, the initial LbL assembly conditions were found to impact the rate of degradation, even when exposing the films to the same degradation conditions. Slight ionization on TA near neutral pH is suggested to cause defects in the interlayer hydrogen-bonding, leaving segments of the polyphosphazene vulnerable to rapid hydrolysis. Finally, the side group chemistry of polyphosphazenes was shown to significantly affect the rate of LbL degradation, with the more hydrophobic hydroxybenzoic acid groups in PCPP slowing the rate of degradation nearly tenfold.

This work on the degradation of polyphosphazene-based LbL films is the first of its kind, to our knowledge, so we envision many future directions for this research, including: validation of the degradation of PYRP-based LbL films in physiological conditions for uses in biomedical applications; substitution of TA with other bioactive hydrogen-bonding molecules; quantifying and further engineering the rate of release of the PYRP side group and loaded bioactive; and inclusion of polyphosphazenes with varied side-group chemistry. Further studies of PYRP-based LbL coatings might lead to highly effective wound treatment or dermal drug delivery systems due to the controlled dual delivery of a skin permeation enhancer and loaded bioactive molecules.

## Data Availability

The data presented in this study are available on request from the corresponding authors.

## Supplementary Materials

The following supporting information can be downloaded at: https://www.mdpi.com/article/10.3390/jfb15020026/s1, Figure S1: PYRP/TA growth curves via QCM; Figure S2: PYRP hydrodynamic diameter over time via DLS; Figure S3: PYRP residual molecular weight over time via AF4; Figure S4: PYRP residual molecular weight over time via SEC; Figure S5: PYRP/TA hydrodynamic diameter over time via DLS; Figure S6: PYRP/TA residual molecular weight over time via AF4; Figure S7: Optical microscope images of as-deposited and degraded TA/PYRP LbL films at pH 3, pH 5, and pH 6.8; Figure S8: Scanning electron micrographs of as-deposited and degraded TA/PYRP LbL films at pH 3, pH 5, and pH 6.8; Figure S9: Infrared shift of the wavenumber for the pyrrolidone carbonyl group in PYRP/TA LbL films assembled and degrading at pH 3; Figure S10: Infrared spectral analysis of pyrrolidone groups (C=O & C-N) compared to the phosphazene group (P-N) for PYRP/TA LbL films assembled and degrading at pH 3; Figure S11: Infrared spectral analysis for PYRP/TA LbL films assembled and degrading at pH 5; Figure S12: Infrared spectral analysis for PYRP/TA LbL films assembled and degrading at pH 6.8; Figure S13: Change in thickness and infrared spectra for PVP/TA LbL films assembled and ‘degrading’ at pH 3; Figure S14: Change in thickness of PYRP/TA LbL films assembled at pH 3 and 6.8, and degrading at pH 5 via spectroscopic ellipsometry; Figure S15: Change in thickness of PCPP/PDADMAC LbL films assembled between pH 6.8 and pH 7.4, and degraded at pH 5 via spectroscopic ellipsometry.
