# Supplementary material for: Engineering Degradation Rate of Polyphosphazene-Based Layer-by-Layer Polymer Coatings
Source: J Funct Biomater. 2024 Jan 23;15(2):26. doi: 10.3390/jfb15020026 (PMC10889497; doi:10.3390/jfb15020026)
Supplement: Supplementary file 1 [file jfb-15-00026-s001.zip › jfb-2791360-supplementary.pdf]

Journal of
Functional Biomaterials

MDPI

Supplementary Materials

Engineering Degradation Rate of Polyphosphazene-Based Layer-by-Layer Polymer Coatings

Jordan Brito [1,†], Junho Moon [1,†], Raman Hlushko [2], Aliaksei Aliakseyeu [3], Alexander K. Andrianov [2,*] and Svetlana A. Sukhishvili [1,*]

[1] Department of Materials Science & Engineering, Texas A&M University, College Station, TX 77840, USA; jordanbrito@tamu.edu (J.B.); jh3@tamu.edu (J.M.)
[2] Institute for Bioscience and Biotechnology Research, University of Maryland, Rockville, MD 20850, USA; hlushkor@umd.edu
[3] Artie McFerrin Department of Chemical Engineering, Texas A&M University, College Station, TX 77843, USA; aliaksei.aliakseyeu@tamu.edu
* Correspondence: andria1@umd.edu (A.K.A.); svetlana@tamu.edu (S.A.S.)
† These authors contributed equally to this work.

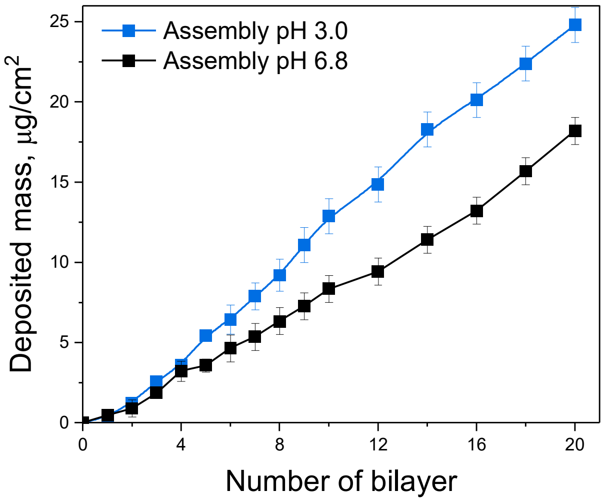

Figure S1. Growth curves of PYRP/TA deposited from pH 3.0 and pH 6.8 solutions, measured using a quartz crystal microbalance (QCM).

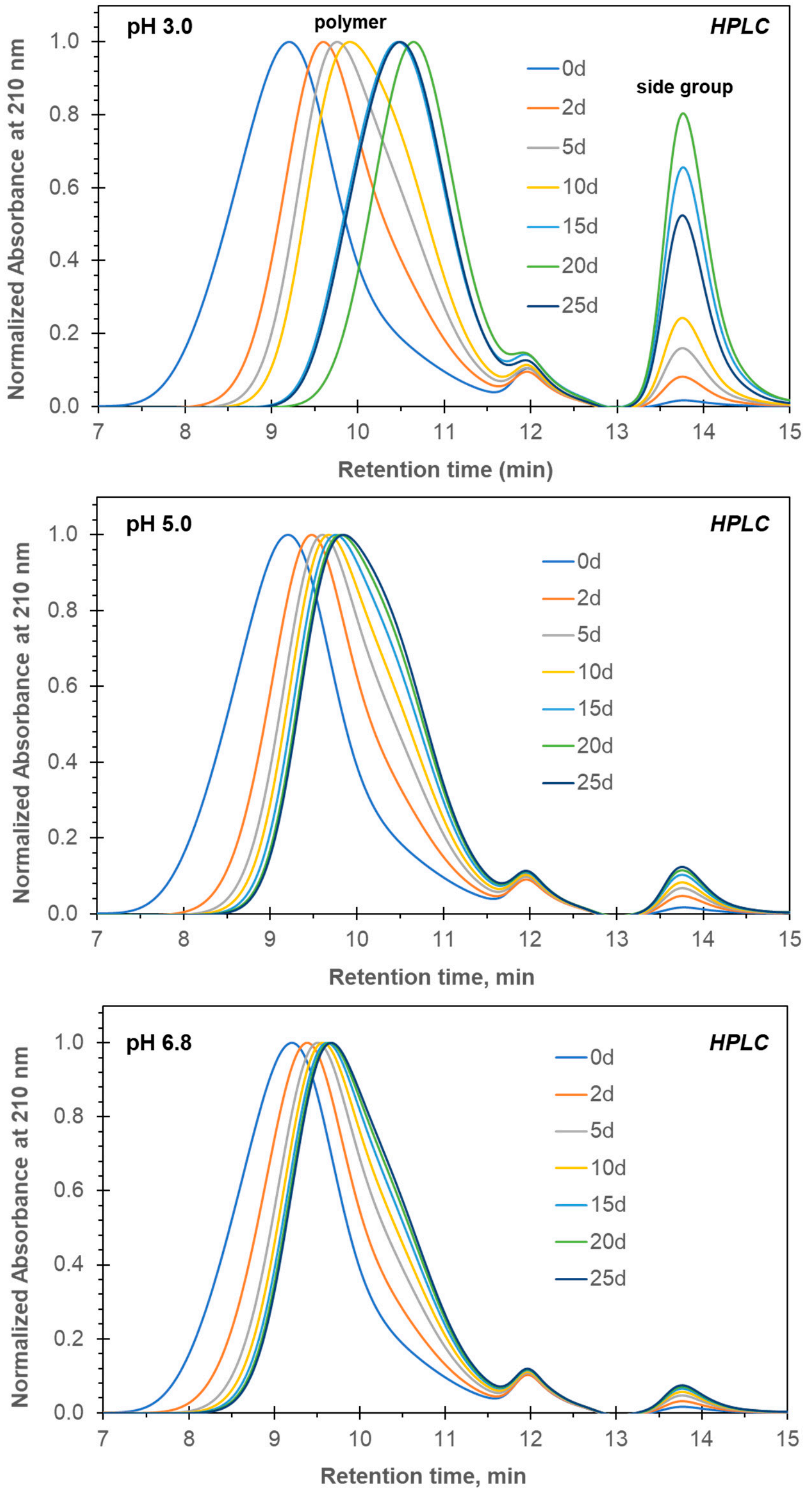

Figure S2. Size-exclusion chromatography (SEC) profiles of PYRP at various time points in solutions at pH 3.0, pH 5.0, and pH 6.8 solution (0.5 mg/mL PYRP, 50 mM phosphate buffer).

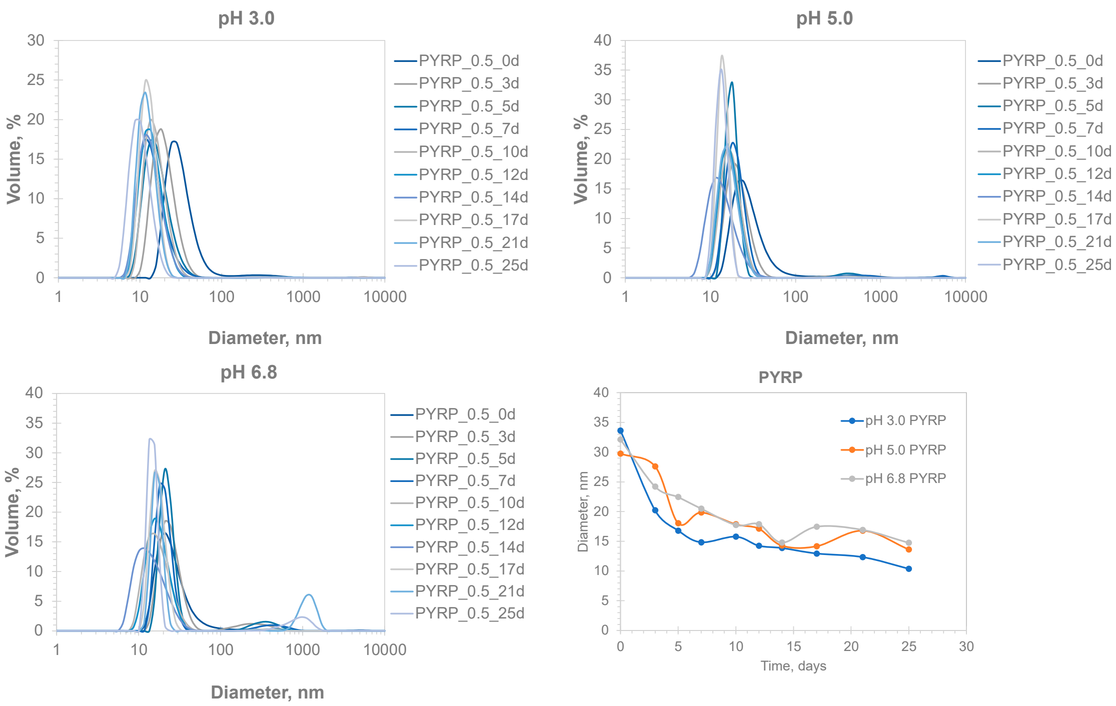

Figure S3. Dynamic light scattering (DLS) profiles of PYRP at various time points and changes in hydrodynamic diameter over time upon exposure to pH 3.0, pH 5.0, and pH 6.8 solution (0.5 mg/mL PYRP, 50 mM phosphate buffer).

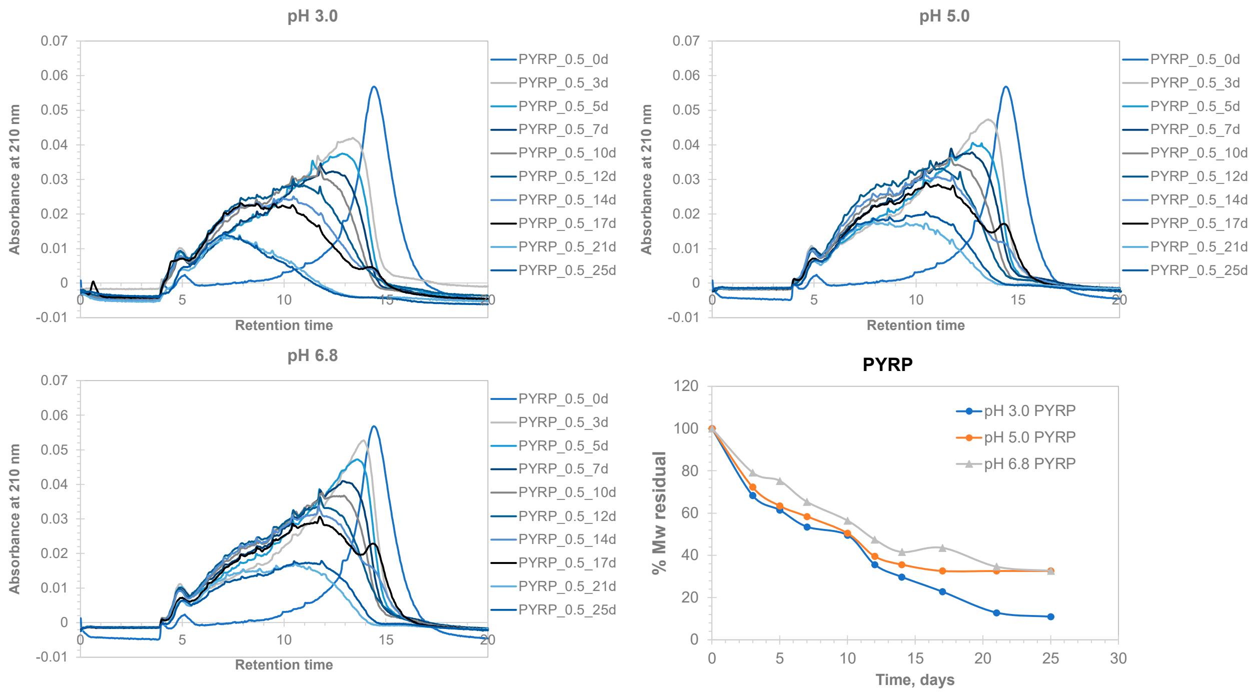

Figure S4. Asymmetric flow field flow fractionation (AF4) profiles at various time points and residual molecular weight of PYRP over time upon exposure to pH 3.0, pH 5.0, and pH 6.8 solution (0.5 mg/mL PYRP, 50 mM phosphate buffer).

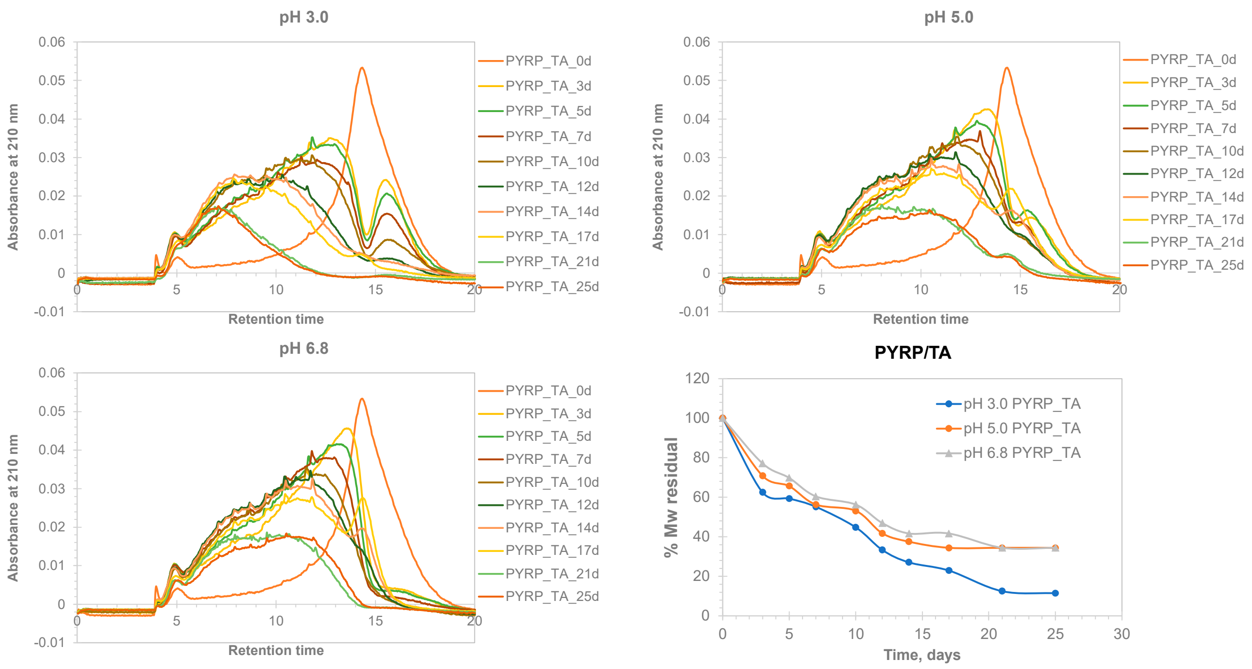

Figure S5. Residual molecular weight of PYRP/TA complexes over time upon exposure to pH 3.0, pH 5.0, and pH 6.8 solution as measured by AF4.

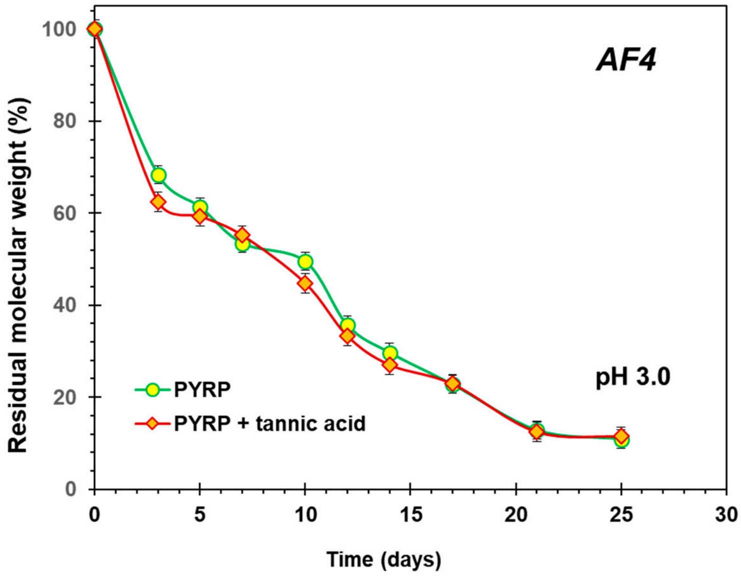

Figure S6. Comparison of PYRP degradation in the absence and presence of tannic acid (contrary to HPLC, AF4 allows to monitor degradation in the presence of tannic acid; however, it does not permit analysis of side group cleavage).

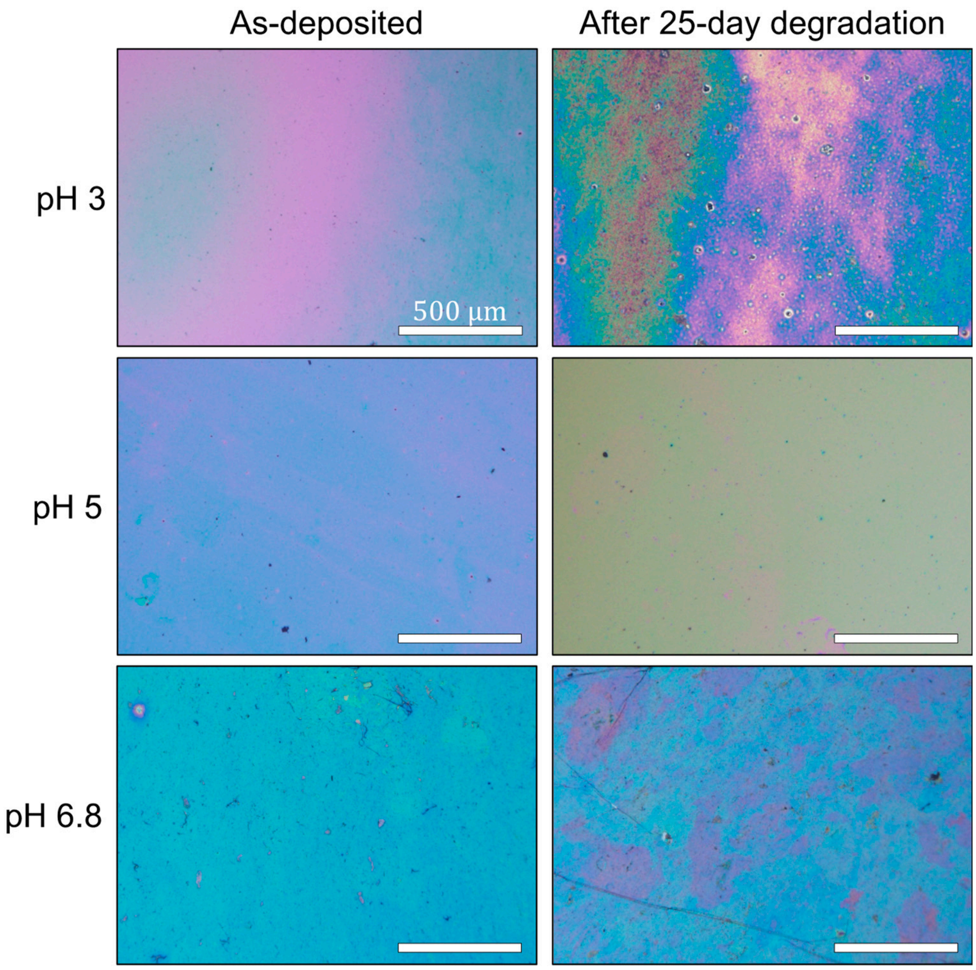

Figure S7. Optical microscope images of 400 nm as-deposited (left column) and 25-day degraded (right column) PYRP/TA LbL films assembled at pH 3, 5, and 6.8, and degraded in solutions of matching pH. The scale bar for all images represents 500 μm.

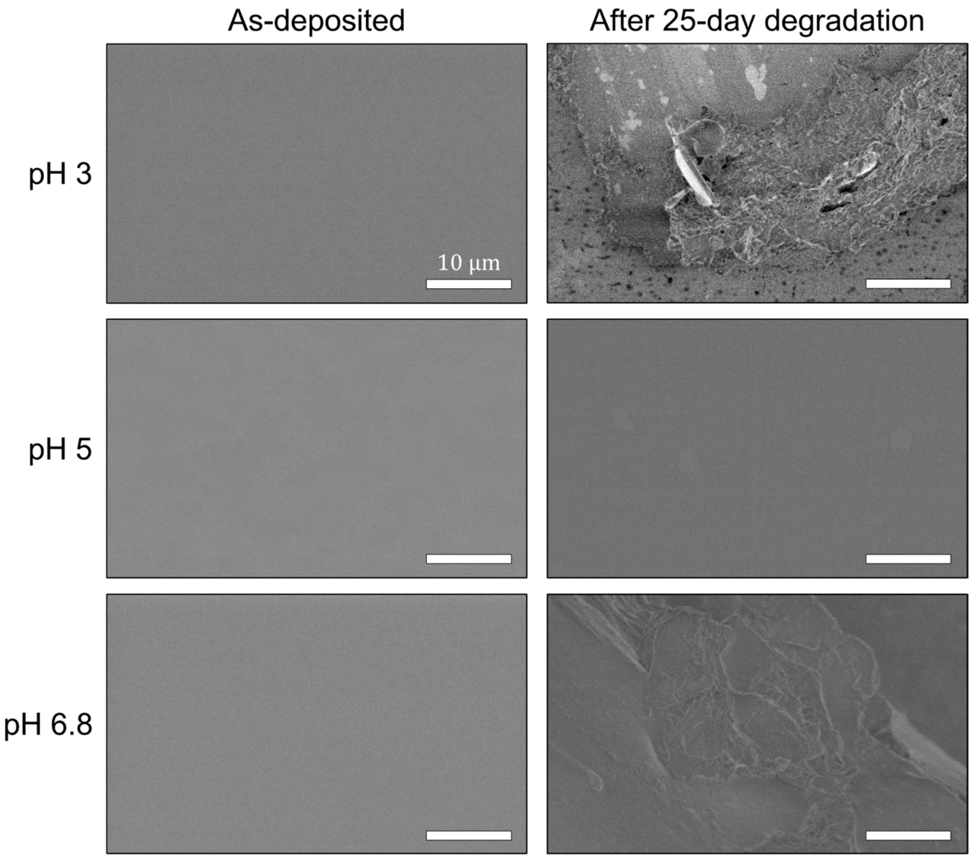

Figure S8. Scanning electron micrographs of 400 nm as-deposited (left column) and 25-day degraded (right column) PYRP/TA LbL films assembled at pH 3, 5, and 6.8, and degraded in solutions of matching pH. The scale bar for all images represents 10 µm.

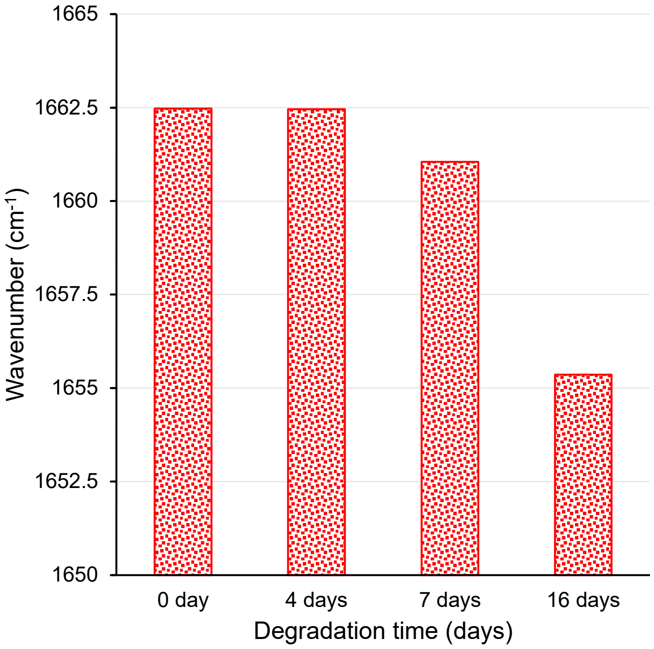

Figure S9. Infrared spectral shift in wavenumber of the pyrrolidone carbonyl group in 400 nm PYRP/TA LbL films assembled at pH 3 and degrading at pH 3.

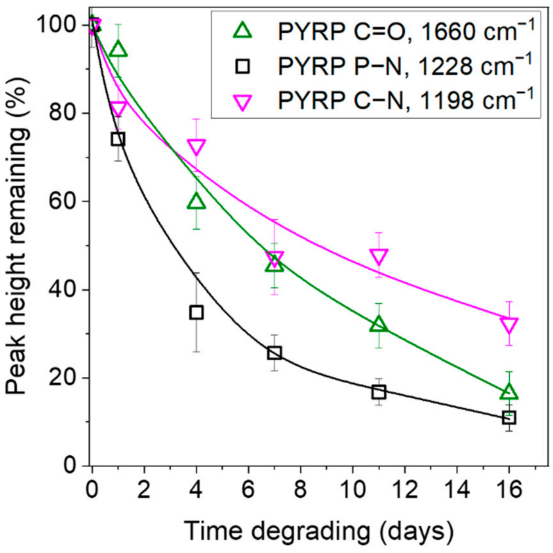

Figure S10. Infrared spectral analysis of pyrrolidone groups (C=O & C-N) compared to the phosphazene group (P-N) in 400 nm PYRP/TA LbL films assembled at pH 3 and degrading at pH 3.

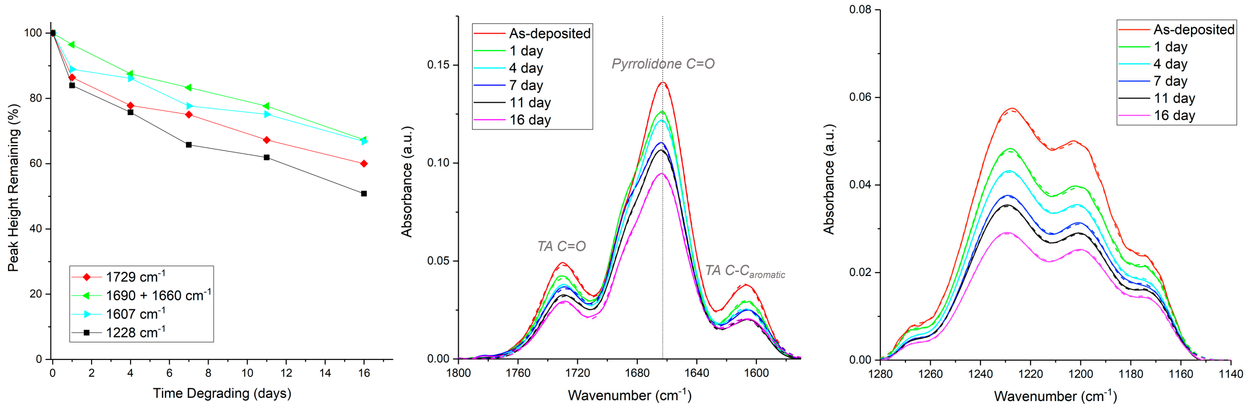

Figure S11. Infrared spectral analysis of pyrrolidone, tannic acid, and phosphazene groups in 400 nm PYRP/TA LbL films assembled at pH 5 and degrading at pH 5.

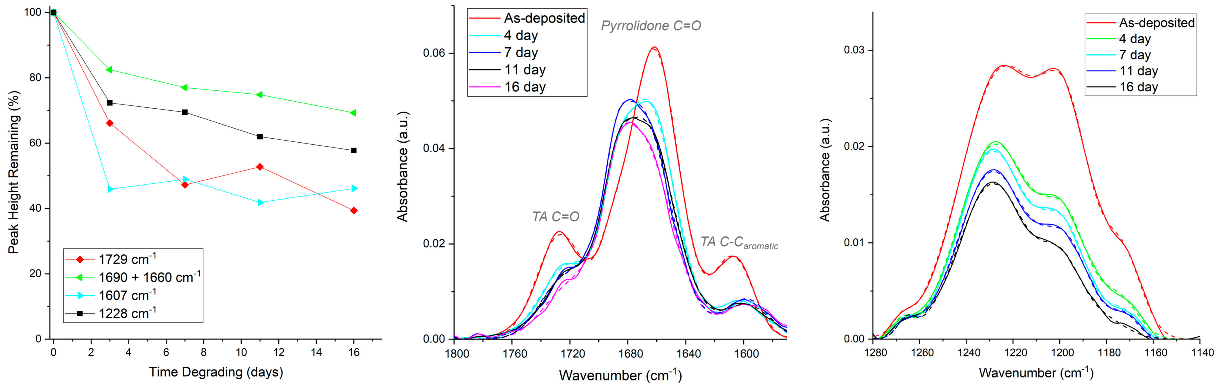

Figure S12. Infrared spectral analysis of pyrrolidone, tannic acid, and phosphazene groups in 400 nm PYRP/TA LbL films assembled at pH 6.8 and degrading at pH 6.8.

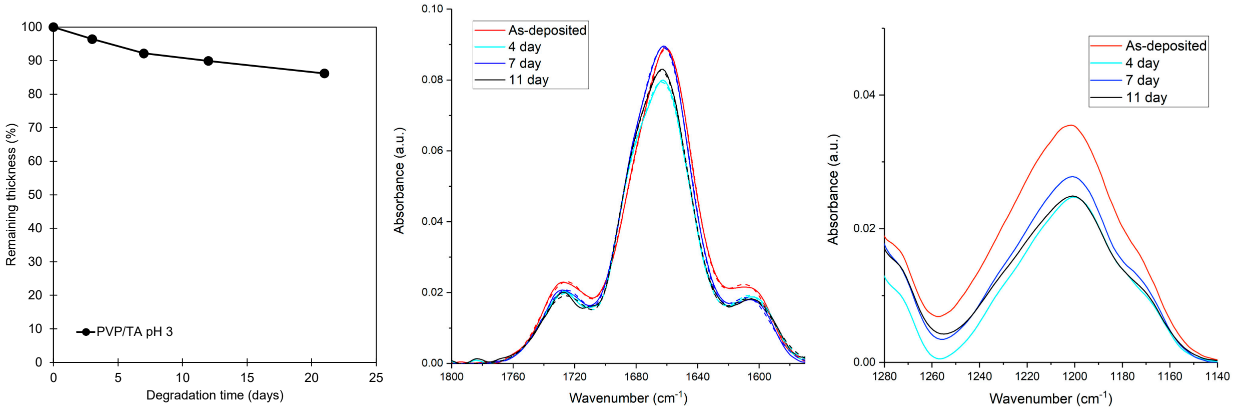

Figure S13. Remaining thickness of 400 nm PVP/TA LbL films assembled at pH 3 and degrading at pH 3, as measured via spectroscopic ellipsometry. Infrared spectra show relative changes in tannic acid (~1725 cm^{-1} & ~1600 cm^{-1}) and pyrrolidone peaks (~1660 cm^{-1} & ~1200 cm^{-1}).

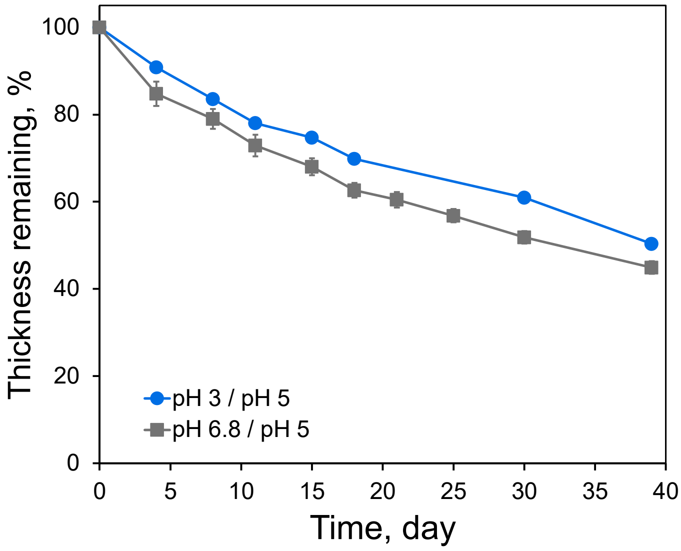

Figure S14. Degradation of 400 nm PYRP/TA LbL films assembled at pH 3 and 6.8 and degrading at pH 5, as determined by spectroscopic ellipsometry.

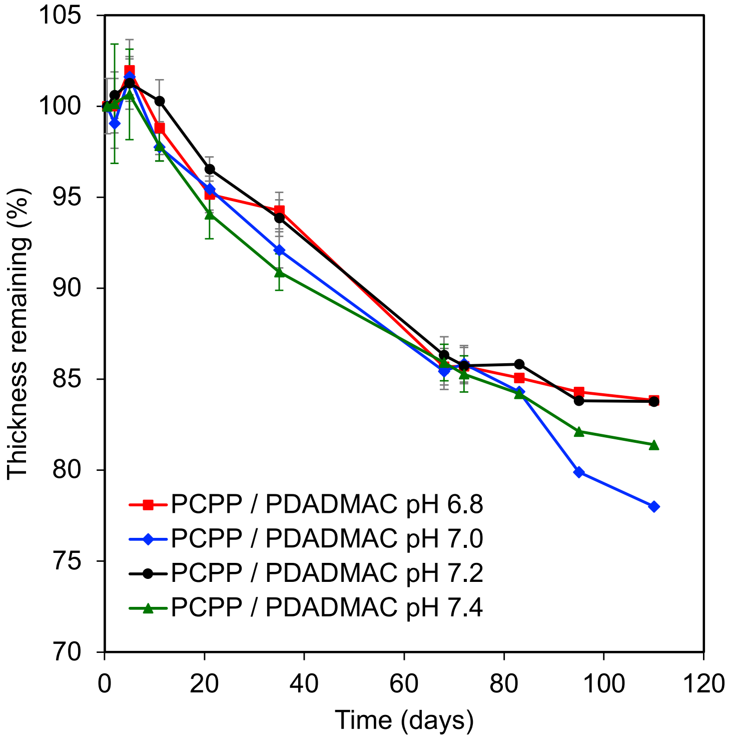

Figure S15. Degradation of 400 nm PCPP/PDADMAC LbL films assembled at pH 6.8, 7.0, 7.2, and 7.4 and degrading at pH 5, as determined by spectroscopic ellipsometry.
